# Biases Inherent in Studies of Coffee Consumption in Early Pregnancy and the Risks of Subsequent Events

**DOI:** 10.3390/nu10091152

**Published:** 2018-08-23

**Authors:** Alan Leviton

**Affiliations:** Boston Children’s Hospital and Harvard Medical School, 1731 Beacon Street, Brookline, MA 02445, USA; alan.leviton@childrens.harvard.edu; Tel.: +1-617-485-7187

**Keywords:** epidemiology, bias, causation, coffee, pregnancy

## Abstract

Consumption of coffee by women early in their pregnancy has been viewed as potentially increasing the risk of miscarriage, low birth weight, and childhood leukemias. Many of these reports of epidemiologic studies have not acknowledged the potential biases inherent in studying the relationship between early-pregnancy-coffee consumption and subsequent events. I discuss five of these biases, recall bias, misclassification, residual confounding, reverse causation, and publication bias. Each might account for claims that attribute adversities to early-pregnancy-coffee consumption. To what extent these biases can be avoided remains to be determined. As a minimum, these biases need to be acknowledged wherever they might account for what is reported.

## 1. Introduction

Maternal consumption of coffee during early pregnancy has been viewed as increasing the risk of miscarriage [1,2,3,4], fetal growth restriction [2,5,6,7,8,9,10,11], and childhood leukemias [12,13,14,15,16,17,18,19,20]. Unfortunately, many of the epidemiologic studies have not acknowledged the potential biases that appear to have influenced these perceptions of risk. The list of potential biases is long [21].

In this essay, I review five of these biases, namely recall bias, misclassification, residual confounding, reverse causation, and publication bias. Each of these biases might account for some of what has been reported. Unfortunately, eliminating these biases can sometimes be extraordinarily difficult, if not impossible. Indeed, a Cochrane Review concluded, “There is insufficient evidence to confirm or refute the effectiveness of caffeine avoidance on birthweight or other pregnancy outcomes. There is a need to conduct high-quality, double-blinded random clinical trials (RCTs) to determine whether caffeine has any effect on pregnancy outcome.” [22]. In essence, observational studies are probably not able to overcome some of the biases. I know of only two clinical trials and they have shown no adverse effect of caffeine consumption on the risk of low birth weight [23], or miscarriage [24]. 

## 2. Bias 1: Recall/Respondent Bias

Recall or respondent bias occurs when the person interviewed does not fully report what is asked or tends to remember the past differently than others. Perhaps the most common form of recall bias occurs when respondents want to present themselves in an idealized light. For example, based on a review of 67 studies that examined the relationship between self-reported smoking and smoking confirmed by cotinine (a metabolite of nicotine) measurement in saliva or urine, the authors concluded, “Overall, the data show trends of underestimation when smoking prevalence is based on self-report.” [25]. Indeed, approximately 20% of pregnant women who report that they are not smokers have smoker-level cotinine concentrations in blood or saliva [26,27]. A review of 34 papers concluded that obese adults tend to significantly under-report their food intake [28]. These reports document that people do not always report the truth. 

One of the explanations offered for much recall/respondent bias is social desirability [29]. As applied to answering questionnaires, social desirability is seen as having two components [30]. One, identified as ‘impression management,’ is the conscious tendency to deceive others, while the other, labeled, ‘self-deception,’ is the unconscious tendency to believe one’s own positive self-reports. Either way, those who try to get accurate information are thwarted by social desirability [31]. whether they want to study hand washing [32], or tobacco consumption [25,33].

Another form of recall bias occurs when some respondents try harder than others to remember the past. For example, when asked to remember exposures during early pregnancy, the mothers of children who developed leukemia are more likely to report higher coffee consumption than the mothers of children selected from the same community, or the mothers of children hospitalized with acute orthopedic trauma [12,13,14,15,16,17,18,19,20,34,35,36]. How well do people remember what they drank years before? The time between the consumption and the query is not the only influence on the accuracy of the information provided. 

Compared to the mothers of healthy newborns, mothers of children with a major congenital malformation diagnosed soon after birth tend to recall more exposures or characteristics during the index pregnancy [37]. This led to the inference that mothers of malformed babies are more likely to try hard to account for what happened than mothers of children who do not have obvious malformations. Preferential recall was also raised by the authors of one study when fathers of children who had leukemia reported levels of cigarette smoking similar to those reported by fathers of ontrols, but mothers of children with leukemia cases reported higher exposure levels to passive smoking than did the mothers of controls [38]. 

The authors of a meta-analysis of studies that evaluated the relationship between maternal coffee consumption and the risk of childhood leukemia acknowledged the possibility that mothers of children who had leukemia might recall exposures during the index pregnancy differently than community controls (“the possibility of a recall bias could not be precluded”) [39]. On the other hand, another meta-analysis “noted the positive association between coffee consumption and childhood ALL and childhood AML among studies using interviewing techniques, but not among studies using self-administrated questionnaire” [40]. The differential recall implies bias somewhere along the information-gathering process. 

In light of these phenomena, strategies to minimize recall bias take several forms. “Cohort studies are generally regarded as providing stronger evidence than case-control studies for causality because they satisfy the temporality criterion that the measurement of exposure precede the ascertainment of the outcome.” [41]. Not surprisingly then, that some tobacco-related exposures (including coffee consumption) are not associated with tobacco-related malignancies in cohort studies (dependent on exposure data collected before recognition of the disorder) [42,43,44], but are reported as associated in case-control studies (dependent on exposure data collected after recognition of the disorder) [45,46]. 

Because of the potential recall bias even when the exposure was recent, some studies of the relationship between caffeine consumption and miscarriage assessed consumption prior to pregnancy [47,48,49,50]. “Overall, while most of these studies were small, the majority showed that pre-pregnancy consumption of caffeine was not associated with increased risk of spontaneous abortion.” [51]. 

One way to minimize recall bias that might have contributed to the association between maternal gestational coffee consumption and childhood leukemias would be to choose controls who also have a potentially fatal illness that might have antenatal origins. This strategy of selecting controls who have another disorder that prompts the mother to search her memory especially thoroughly [52], has yet to be applied to the study of childhood leukemia. It would be reasonable to do so if the malignancies of controls each had a relatively unique risk profile. 

## 3. Bias 2: Misclassification

The most obvious misclassification that has the potential to distort our perception of truth about relationships between coffee drinking and any disorder is inappropriately quantifying exposure [53]. What is a cup of coffee? 5 ounces (150 mL)? 8 ounces (240 mL)? Is a mug 8 ounces (240 mL)? 10 ounces (300 mL)? 12 ounces (360 mL)? Similar concerns apply to the ‘strength of the brew’, as well as to additives (e.g., sugar, non-nutritive sweeteners, milk, cream).

Misclassification bias is potentially high in studies that assess the effects of caffeine as the exposure of interest. Almost invariably, authors make assumptions based on reports of caffeine content of coffee, tea, other beverages and foods [54,55,56], and about attributing to a population, the caffeine content as estimated by self-report [57,58].

## 4. Bias 3: Residual Confounding

Confounding defines the distortion of our perception of the relationship between an exposure (coffee consumption) and a disorder (e.g., childhood leukemia, miscarriage). 

This distortion occurs when a variable that is a potential confounder is not considered in the analysis. A potential confounder has to be associated with the disorder and the exposure, but must not be on the causal pathway between the exposure and the disorder [59].

Tobacco smoke induces cytochrome P450 1A2 (CYP1A2), the main enzyme involved in caffeine metabolism, thereby increasing the rate of caffeine metabolism, and shortening the half-life of caffeine [60,61,62]. One consequence is that the duration of desired behavioral effects of caffeine is shortened, prompting smokers to consume more coffee than non-smokers [63]. Among Norwegian pregnant women, the average daily caffeine consumption varied with smoking. For example, never-smokers consumed 54 mg of caffeine daily, while occasional smokers consumed 109 mg daily, and daily smokers consumed on average 143 mg each day [8]. Therefore, tobacco is a potential confounder of the relationship between a mother’s coffee consumption and her child’s risk of childhood leukemia. This can be minimized to some extent by “adjusting” for tobacco exposure. 

Residual confounding occurs when efforts to minimize confounding are not adequate. 

In the most extreme examples, investigators classify as “smokers” all women who smoked during pregnancy, even though these women varied considerably in their level of tobacco consumption, and classify all others as “non-smokers”. 

Tobacco smoke exposure is a known carcinogen [64]. Some studies have reported that maternal tobacco exposure during pregnancy is associated with increased risk of the offspring developing childhood leukemia [13,19,65,66,67], whereas others report that paternal tobacco exposure during pregnancy (a source of second-hand smoke for the mother) is associated with the child’s heightened risk of childhood leukemia [34,38,68].

Successful adjustment in multivariable models of the risk of a disorder depends on high-quality exposure data. All the adjusting in the world cannot eliminate distortions due to “social desirability responding,” such as that which occurs when respondents are truthful about their coffee consumption, but not about their tobacco exposure. 

The statement, “Only at the very highest level of pre-pregnancy intake (e.g., >900 mg/day, a consumption level rarely seen in people who do not smoke) was caffeine consumption associated with increased risk of miscarriage” [49]. This raises the possibility that such high consumptions reflect the influence of tobacco exposure, which leads to the inference that the increased risk of miscarriage might also reflect residual confounding of tobacco [69]. Cigarette smoking is also a risk factor for low birth weight [70] and placenta dysfunction [71]. These associations again raise the possibility of residual confounding in studies of coffee consumption during early pregnancy and low birth weight [8,72,73], fetal growth restriction [74], and perhaps even epigenetic effects, such as childhood overweight [75]. 

Another challenge to eliminating confounding is posed by polymorphisms of multiple genes that influence caffeine and/or coffee consumption [76,77,78,79,80,81,82,83,84]. Some of these polymorphisms also influence the risk of diseases associated with caffeine and/or coffee consumption [85,86,87,88,89,90]. 

A common strategy to disentangle the contribution of genetic propensity to consume coffee/caffeine is to stratify the sample by possession of each gene variant. In essence, this amounts to exploring the caffeine/coffee association in those with and without a specific variant. However, this can be considerably more complex and pose analysis challenges. For example, alleles near genes associated with high coffee consumption are associated with adiposity, cigarette smoking, high levels of fasting insulin and glucose, low risk of hypertension, as well as favorable lipid, inflammatory, and liver enzyme profiles [82]. 

## 5. Bias 4: Reverse Causation 

### 5.1. Coffee Consumption Changes during Early Pregnancy

Even before some women realize they are pregnant, they decrease their coffee consumption. Coffee consumption by women tends to decline as early as the 4th and 5th weeks of normal pregnancy [91,92] (see Figure 1 of [91]; and Figures 1 and 2 of [92]). 

Perhaps the first signal of a viable pregnancy is the sensitivity to odors, which can be accompanied by a diminished desire for coffee and the aromas associated with it [93]. As the pregnancy signal intensifies, nausea and overt aversion to odors become increasingly evident [94]. 

Because women who have early nausea are at a lower risk of early fetal loss (miscarriage) than women who do not experience nausea [95,96,97], a strong pregnancy signal is seen as an indicator of a viable pregnancy, and the absence of a pregnancy signal is seen as an indicator that the situation might be suboptimal.

The decline in coffee consumption early in pregnancy among women who apparently did not intend to reduce their coffee consumption has been attributed to epiphenomena, including “aversion to tastes and smells ordinarily well tolerated.” [98]. Subsequently, the term “pregnancy signal” was used to describe some of the earliest physiologic changes associated with pregnancy, including food aversions, and (hyper)sensitivities to aromas, including those of brewed coffee and perfume [99,100,101,102]. Some now use the term, ‘pregnancy awareness’ [103].

More than half a century ago, the pregnancy signal was attributed to the high-estrogen-content of the first commercially-available oral contraceptives [104,105]. Two decades later, the pregnancy signal was linked to elevated (early morning) urine concentrations of estrone-3-glucuronide and human chorionic gonadotropin [106]. “The number of potential contributors to maternal recognition of pregnancy continues to grow and this highlights our limited appreciation of the complexity of the key molecules and signal transduction pathways that intersect during these key developmental processes.” [107]. And indeed, the number of potential contributors does continue to grow [108].

The hormonal characteristics linked to coffee consumption during pregnancy to some extent also appear to apply to consumption when women are not pregnant. For example, the lower the peak estradiol level among women prior to in vitro fertilization, the higher their caffeine consumption [109]. A similar phenomenon occurs in premenopausal women [110].

### 5.2. Inferences That Follow from a Weak Pregnancy Signal

If a weak pregnancy signal is an indicator of a placenta not able to produce the high concentrations of hormones and growth factors needed for fetal wellbeing and optimal growth, the fetus is at increased risk of death and limited growth. If a weak pregnancy signal also allows the gravida to continue her normal coffee consumption, then coffee will be blamed (inappropriately) for increasing the risk of miscarriage and lower birth weight. The blame is inappropriate because the level of coffee consumption is influenced by the very process that will result in potentially dire consequences. In essence, the same placental deficiencies that contribute to the adversities also fail to reduce coffee consumption. Continued pre-pregnancy level of coffee consumption is a consequence, and not a cause, of the placental deficiencies. 

This is an example of “reverse causation,” which refers to situations where an antecedent is a consequence rather than a cause of illness [50,111,112,113,114,115,116,117,118,119,120,121,122,123,124,125,126,127,128]. Another example has occurred in some studies that have found that people whose weight (or body mass index) is low were at heightened risk of death [129]. Low weight can be a consequence of disease that results in a loss of appetite [130]. In such situations the processes that lead to death also lead to weight loss, rather than low weight contributing to mortality risk [131]. 

“Reverse causation” also applies to the situation where a limited-function placenta is more likely to allow a woman to continue her usual levels of coffee consumption throughout pregnancy than is the healthy placenta that prompts a woman to reduce her coffee consumption. As a result, coffee consumption is associated with the consequences of a limited-function placenta precisely because a limited-function placenta allows higher coffee consumption than does a full-function placenta. 

The limited-function placenta is associated with fetal growth restriction [132,133,134]. So is coffee/caffeine consumption [9], even if only by reverse causation. 

Among the risk factors for childhood leukemias are two pregnancy phenomena, prior pregnancy loss (“fetal wastage”) [135,136,137,138] and low birth weight [139,140,141,142,143]. Both of these have been associated with continued normal (pre-pregnancy) level of coffee consumption [6,8,11,91,144,145,146,147]. To some extent, each of these (i.e., fetal wastage, low birth weight, and continued coffee consumption during pregnancy at pre-pregnancy levels) is a correlate of impaired implantation of the placenta, and a weaker pregnancy signal than occurs following a healthy implantation. Might the association between gestational coffee consumption and childhood leukemia reflect “reverse causation”? 

## 6. Bias 5: Publication Bias 

Publication (or dissemination) bias has been defined as the selective publication of studies [148,149]. This appears to happen most commonly when reviewers and editors view “positive” findings as more attractive for publication than “negative” (or non-significant) findings [150]. Publication bias can also reflect self-censorship by authors who are reluctant to continue to battle editors about the need to publish reasonably-powered “negative” studies [151]. So many other persistent influences contribute to publication bias [152,153,154] that some do not consider elimination of this bias to be feasible [155,156]. Consequently, a negative finding, such as no relationship between early pregnancy coffee consumption and risk of miscarriage, is unlikely to be attractive to editors in light of the plethora of studies reporting a positive relationship. The result is publication bias [157,158], which is especially distorting in meta-analyses [159]. These include distortion of the standardized mean difference plotted against the standard error, which can be severe when the primary studies are small [160]. In addition, asymmetry of the funnel plot might not accurately indicate publication bias [161].

## 7. Conclusions

I have provided comments about biases that might account for associations between maternal coffee consumption early in pregnancy and subsequent events. All of the reports of detrimental effects of coffee consumption during early pregnancy can be explained by one or more of the biases mentioned above. To what extent these biases explain away the associations between maternal early-pregnancy coffee consumption and subsequent events remains to be determined. Obviously, the more these biases can be avoided, the closer we will come to the truth. A laudable goal, but one that is difficult to achieve.
